# Correction: Transcriptome analysis reveals Nitrogen deficiency induced alterations in leaf and root of three cultivars of potato (*Solanum tuberosum* L.)

**DOI:** 10.1371/journal.pone.0253994

**Published:** 2021-06-24

**Authors:** Jingying Zhang, Yaping Wang, Yanfei Zhao, Yun Zhang, Jiayue Zhang, Haoran Ma, Yuzhu Han

There are errors in the Funding statement. The correct Funding statement is as follows: This research was funded by the project of Science and Technology Department of Jilin Province in the form of a grant (20200301025RQ). The funders had no role in study design, data collection and analysis, decision to publish, or preparation of the manuscript.
