# Supplementary material for: Transcriptome analysis reveals Nitrogen deficiency induced alterations in leaf and root of three cultivars of potato (Solanum tuberosum L.)
Source: PLoS One. 2020 Oct 29;15(10):e0240662. doi: 10.1371/journal.pone.0240662 (PMC7595393; doi:10.1371/journal.pone.0240662)
Supplement: S1 Table — (DOCX) [file pone.0240662.s001.docx]

Table S1. The sequences of primers used for the qRT-PCR analysis.

| Primer | Forward Primer | Reverse Primer | Tm |
| --- | --- | --- | --- |
| EF1α | GATGGTCAGACCCGTGAACA | CCTTGGAGTACTTCGGGGTG | 58 |
| NRT2.4 (PGSC0003DMG400001145) | GGAATATGGGTGGTGGTGCT | TCTCCAAGCAGTAAACGGGG | 58 |
| NRT2.5 (PGSC0003DMG400016996) | TCGTTCGTTTCTTCACGGGG | CCACCAAGATTCCCCCATCC | 58 |
| NRT2.7 (PGSC0003DMG400006913) | GGATGAGAGGGCGGTTATGG | AACCAGAACAGACGCCCAAA | 58 |
| NR (PGSC0003DMG400030212) | ATTCCGGGATTCATCGGTGG | CACCATGCTTCAGCGTTAGC | 58 |
| NiR (PGSC0003DMG400008262) | TGTAACGACGAGTGCCCAAA | AACCCCACGAATCTGCCAAT | 58 |
| NiR (PGSC0003DMG400025823) | ACATTCCAGTGGGTCGTGTC | CACAGTCAGCCGTAGCTCTC | 58 |
| GdH (PGSC0003DMG400016001) | ATGTCCCTGCGCCTGATATG | TTCCCTACCCAATGAACCGC | 58 |
| GdH (PGSC0003DMG400008356) | CAGCGGTCGCCAATATACCA | TGCAGGAACATCGGTGTGAA | 58 |
| GS (PGSC0003DMG400004355) | CTACTGTGGTGCTGGAGTGG | TCCTGGCATAACCTCTCCGT | 58 |
| GS (PGSC0003DMG400014592) | GGTGAAGGCAATGAACGTCG | CTCTGCCTACACGAACCGAG | 58 |
| Fd-GOGAT (PGSC0003DMG400009698) | CAACGTGGTGCCAGTAATGC | TTGCGATAAAACCGACCCCA | 58 |
| NADH-GOGAT (Novel02273) | ATTGAAGAGGCCCACCCAAG | TGTAAGACACACCCTGTCGC | 58 |
