# Supplementary material for: Transcriptome analysis reveals Nitrogen deficiency induced alterations in leaf and root of three cultivars of potato (Solanum tuberosum L.)
Source: PLoS One. 2020 Oct 29;15(10):e0240662. doi: 10.1371/journal.pone.0240662 (PMC7595393; doi:10.1371/journal.pone.0240662)
Supplement: S2 Table — (DOCX) [file pone.0240662.s002.docx]

Table S2. RNA-seq data summary and quality analysis.

| Sample name | Raw reads | Clean reads | clean bases (G) | Q30(%) | GC content(%) |
| --- | --- | --- | --- | --- | --- |
| YNa1 | 43553516 | 40452950 | 6.07 | 92.71 | 40.81 |
| YNa2 | 49397496 | 47125288 | 7.07 | 93.3 | 41.32 |
| YNa3 | 46372144 | 43760828 | 6.56 | 92.83 | 40.97 |
| Ya1 | 46844314 | 43120680 | 6.47 | 92.72 | 41.57 |
| Ya2 | 48279028 | 45006728 | 6.75 | 92.87 | 41.52 |
| Ya3 | 50000852 | 47654700 | 7.15 | 92.64 | 41.46 |
| YNb1 | 47910752 | 45964542 | 6.89 | 95.28 | 41.47 |
| YNb2 | 41701132 | 39590930 | 5.94 | 94.14 | 41.18 |
| YNb3 | 50061142 | 46859126 | 7.03 | 92.67 | 41.67 |
| Yb1 | 47884150 | 46136544 | 6.92 | 95.06 | 40.87 |
| Yb2 | 55811914 | 52188706 | 7.83 | 92.76 | 40.85 |
| Yb3 | 44245698 | 42300578 | 6.35 | 92.58 | 40.71 |
| CNa1 | 50373138 | 47667946 | 7.15 | 95.31 | 40.94 |
| CNa2 | 52777698 | 48984518 | 7.35 | 92.27 | 41.01 |
| CNa3 | 42031206 | 39195826 | 5.88 | 92.94 | 41.15 |
| Ca1 | 52640248 | 49063908 | 7.36 | 92.81 | 40.9 |
| Ca2 | 44863940 | 42847098 | 6.43 | 95.05 | 42.24 |
| Ca3 | 45933510 | 43394476 | 6.51 | 92.65 | 40.79 |
| CNb1 | 51434832 | 48259582 | 7.24 | 92.75 | 42.03 |
| CNb2 | 46581212 | 44516670 | 6.68 | 95.06 | 41.42 |
| CNb3 | 43905748 | 40982774 | 6.15 | 93.81 | 41.15 |
| Cb1 | 43734128 | 41505994 | 6.23 | 93.79 | 41.53 |
| Cb2 | 45860162 | 43038040 | 6.46 | 93.95 | 41.3 |
| Cb3 | 45879658 | 43563268 | 6.53 | 95.09 | 41.23 |
| XNa1 | 46899232 | 43769300 | 6.57 | 94.23 | 41.96 |
| XNa2 | 57345042 | 53232254 | 7.98 | 95.43 | 41.12 |
| XNa3 | 46750862 | 44598066 | 6.69 | 95.08 | 42.11 |
| Xa1 | 47558180 | 44671068 | 6.7 | 94.34 | 41.54 |
| Xa2 | 43038682 | 41191286 | 6.18 | 95.18 | 41.53 |
| Xa3 | 47776386 | 44849112 | 6.73 | 93.9 | 42.03 |
| XNb1 | 42431652 | 40343416 | 6.05 | 93.68 | 42.07 |
| XNb2 | 47437900 | 44463660 | 6.67 | 95.28 | 41.43 |
| XNb3 | 53384992 | 51431704 | 7.71 | 94.93 | 41.89 |
| Xb1 | 44452622 | 41796432 | 6.27 | 93.84 | 41.83 |
| Xb2 | 46259924 | 43649738 | 6.55 | 93.62 | 42.25 |
| Xb3 | 45425822 | 42613572 | 6.39 | 93.8 | 42.2 |
| Total/Average | 1627.77 M | 1535.22 M | 241.29 | 93.84 | 41.45 |
