# Supplementary material for: Transcriptome analysis reveals Nitrogen deficiency induced alterations in leaf and root of three cultivars of potato (Solanum tuberosum L.)
Source: PLoS One. 2020 Oct 29;15(10):e0240662. doi: 10.1371/journal.pone.0240662 (PMC7595393; doi:10.1371/journal.pone.0240662)
Supplement: S3 Table — (DOCX) [file pone.0240662.s003.docx]

Table S3. Several key enzymes differentially expressed by N deficiency and between cultivars and associated with the N metabolism.

| No. | KO /Entry number | Gene ID (name) | Gene annotation | Metabolic pathways |
| --- | --- | --- | --- | --- |
| 1 | Sot:102584553 K02575 | PGSC0003DMG400015734 | MFS transporter, NNP family, nitrate/nitrite transporter (high affinity nitrate transporter 2.4-like) | Nitrogen metabolism  (Nitrate assimilation) |
| 2 | Sot:102596700 K02575 | PGSC0003DMG400001145 | MFS transporter, NNP family, nitrate/nitrite transporter  (high affinity nitrate transporter 2.4-like) |  |
| 3 | Sot:102588784 K02575 | PGSC0003DMG402011998 | MFS transporter, NNP family, nitrate/nitrite transporter（high affinity nitrate transporter 2.4-like） |  |
| 4 | sot:102583246 | Novel03457 | MFS transporter, NNP family, nitrate/nitrite transporter  (high affinity nitrate transporter 2.4-like) |  |
| 5 | Sot:102581584 K02575 | PGSC0003DMG400019674 | MFS transporter, NNP family, nitrate/nitrite transporter  (high affinity nitrate transporter 2.4-like) |  |
| 6 | Sot:102586376 K02575 | PGSC0003DMG400016996 | MFS transporter, NNP family, nitrate/nitrite transporter  (high affinity nitrate transporter 2.5) |  |
| 7 | Sot:102590802 K02575 | PGSC0003DMG400006913 | MFS transporter, NNP family, nitrate/nitrite transporter  (high affinity nitrate transporter 2.7) |  |
| 8 | Sot:102577728  K10534 | PGSC0003DMG400030212 | Nitrate reductase (NAD(P)H) [EC:1.7.1.1 1.7.1.2 1.7.1.3]  (NADH nitrate reductase) | Nitrogen metabolism  (Assimilatory nitrate reduction, nitrate ammonia; Nitrate assimilation) |
| 9 | Sot:102581851  K00366 | PGSC0003DMG400025823  (NIR) | Ferredoxin-nitrite reductase [EC:1.7.7.1]  (ferredoxin: nitrite reductase, chloroplastic) |  |
| 10 | sot:102596437  K00366 | PGSC0003DMG400008262 | Ferredoxin-nitrite reductase [EC:1.7.7.1]  (ferredoxin--nitrite reductase, chloroplastic-like) |  |
| 11 | Sot:102591839  K01455 | PGSC0003DMG400024965 | Formamidase [EC:3.5.1.49]  (formamidase-like) | Cyanoamino acid metabolism; Glyoxylate and dicarboxylate metabolism; Nitrogen metabolism; |
| 12 | sot:102589383  K01674 | Novel00952 | carbonic anhydrase [EC:4.2.1.1]  (alpha carbonic anhydrase 7-like) | Nitrogen metabolism |
| 13 | sot:102589374  K01673 | PGSC0003DMG400006956;  PGSC0003DMG400006957 | carbonic anhydrase [EC:4.2.1.1]  (carbonic anhydrase, chloroplastic-like) |  |
| 14 | sot:102602780  K01674 | PGSC0003DMG400008954 | carbonic anhydrase [EC:4.2.1.1]  (alpha carbonic anhydrase 1, chloroplastic) |  |
| 15 | sot:102604042  K01673 | PGSC0003DMG400030984 | carbonic anhydrase [EC:4.2.1.1] (carbonic anhydrase 2) |  |
| 16 | sot:102581352  K01674 | Novel01254 | carbonic anhydrase [EC:4.2.1.1] (alpha carbonic anhydrase 7-like) |  |
| 17 | sot:102581683  K01674 | PGSC0003DMG400008094 | carbonic anhydrase [EC:4.2.1.1] (alpha carbonic anhydrase 7-like) |  |
| 18 | sot:102599109  K01673 | PGSC0003DMG400008827 | carbonic anhydrase [EC:4.2.1.1] (beta carbonic anhydrase 5, chloroplastic) |  |
| 19 | sot:102591490  K01673 | PGSC0003DMG400000493 | carbonic anhydrase [EC:4.2.1.1] (carbonic anhydrase, chloroplastic） |  |
| 20 | sot:102578903  K01725 | PGSC0003DMG400017160 | cyanate lyase [EC:4.2.1.104] (cyanate hydratase) |  |
| 21 | sot:102585678  K00261 | PGSC0003DMG400008356 | glutamate dehydrogenase (NAD(P)+) [EC:1.4.1.3]  ( glutamate dehydrogenase) | Arginine biosynthesis;  Alanine, aspartate and glutamate metabolism;  Nitrogen metabolism; Metabolic pathways;  Carbon metabolism |
| 22 | sot:102586580  K00261 | PGSC0003DMG400016001 | glutamate dehydrogenase (NAD(P)+) [EC:1.4.1.3]  (glutamate dehydrogenase A-like) |  |
| 23 | Sot:102589055  K00261 | PGSC0003DMG400027589 | glutamate dehydrogenase (NAD(P)+) [EC:1.4.1.3]  (glutamate dehydrogenase A-like) |  |
| 24 | Sot:102599312  K00261 | PGSC0003DMG400008344 | glutamate dehydrogenase (NAD(P)+) [EC:1.4.1.3]  ( glutamate dehydrogenase A) |  |
| 25 | Sot:102595103  K01915 | PGSC0003DMG400023620 | glutamine synthetase [EC:6.3.1.2]  (glutamine synthetase cytosolic isozyme 1-1) | Arginine biosynthesis ;  Alanine, aspartate and glutamate metabolism ;  Glyoxylate and dicarboxylate metabolism ;  Nitrogen metabolism ; Metabolic pathways ;  Biosynthesis of amino acids |
| 26 | sot:102603663  K01915 | Novel00186, PGSC0003DMG400004355 | glutamine synthetase [EC:6.3.1.2]  (glutamine synthetase, chloroplastic-like) |  |
| 27 | sot:102596495  K01915 | PGSC0003DMG400014592 | glutamine synthetase [EC:6.3.1.2]  (glutamine synthetase-like) |  |
| 28 | sot:102596783  K01915 | PGSC0003DMG400013235 | glutamine synthetase [EC:6.3.1.2]  (glutamine synthetase) |  |
| 29 | sot:102580516  K00264 | Novel02273 | glutamate synthase (NADPH/NADH) [EC:1.4.1.13 1.4.1.14]  (glutamate synthase 1 [NADH], chloroplastic) |  |
| 30 | sot:102600112  K00284 | PGSC0003DMG400009698 | glutamate synthase (ferredoxin) [EC:1.4.7.1]  ( ferredoxin-dependent glutamate synthase, chloroplastic) |  |
