# Supplementary material for: Transcriptome analysis reveals Nitrogen deficiency induced alterations in leaf and root of three cultivars of potato (Solanum tuberosum L.)
Source: PLoS One. 2020 Oct 29;15(10):e0240662. doi: 10.1371/journal.pone.0240662 (PMC7595393; doi:10.1371/journal.pone.0240662)
Supplement: Attachment — Submitted filename: Point-by-point response.docx [file pone.0240662.s004.docx]

**Dear Editor,**

Thank you for your letter and for the reviewers’ comments concerning our manuscript entitled “Nitrogen deficiency induced alterations in leaf and root of three cultivars of potato (Solanum tuberosum L.)”. Those comments are all valuable. We have studied comments carefully and made corrections.

The revised manuscript is highlighted in Tracked Changes version.

**Point-by-point response to the reviewer’s comments**

Reviewer reports:

**Reviewer 1:**

The study presents unique and original data that will significantly contribute to our understanding on the molecular underpinnings of nitrogen responses in plants particularly on potato. While it has already been reported by Jozefowicz et al. (2017), nitrogen deficiency does induce alterations in root proteome of potato varieties contrasting their response to low N. Just recently, Tiwari et al. (2020) presented their results on the transcriptome analysis of potato shoots, roots, and stolons under nitrogen stress. This paper by Zhang et al. investigates the association of transcriptomic profiles with the efficiencies of N using 3 potato cultivars. This study will have a bigger impact if the authors discuss the implications of their results in relation to genetic variability.

Unfortunately, the paper is poorly written. Presentation of ideas and concepts are hard to follow as it lacks the logical flow for the entire manuscript to be comprehensible. Although the experiments and statistical analysis have indeed been performed to high technical standard, however, further analysis of their data are still needed and presented for discussion and elaborated in their conclusion. Unfortunately, the conclusion is barely comprehensible as it only reiterates their findings without discussing any implications.

**Response: Thank you for your comments. Firstly, we accept the suggestion to try our best to improve the English language. To further improve the quality of written English, the manuscript has also been edited by Caughman Corey, who was a native English speaker. Then, we have reorganized the manuscript and hope that the new manuscript will be more logical. In addition, to better present the results, we have made major revisions to the results section. New data were added. Also, we re-drawn figure 3 and added N-related gene expression results (Figure 4). Importantly, we have revised the discussion and conclusion sections, hoping that the newly submitted manuscript will be improved.**

Also, may I suggest the title to include the term “Transcriptome” as the entire paper revolves around transcriptomic analysis of potato cultivars under nitrogen deficiency.

**Response: Thank you for your comments. We re-titled the manuscript to “Transcriptome analysis reveals Nitrogen deficiency induced alterations in leaf and root of three** **cultivars of potato (Solanum tuberosum L.)”**

**Reviewer 2:**

First of all, I suggest to the authors a substantial revision of the English, since there are many spelling mistakes in the text. I could not make corrections of all of them. I suggest to the author to send the manuscript to a native English speaker before submission.

**Response: Thank you for your comments. We accept the suggestion to try our best to improve the English language. To further improve the quality of written English, the manuscript has also been edited by Caughman Corey, who was a native English speaker.**

At the LINE 21 – 27 of the ABSTRACTS, there is a simple list of methods used in the work, without any results and discussion. Moreover, is not clear what authors mean with the sentence “and root were detected” (LINE 23). I suggest to re-write the abstract, highlighting better the results. In the present version, it is too descriptive.

**Response: Thank you for your comments. We re-write the abstract section and highlight the main results. The reorganized abstract is improved.**

The INTRODUCTION is well written, and rigidly follows the discussion of some important issues. It initially introduces the importance of potato from a nutritional and agronomic point of view, and then discusses into details the main problems linked to the cultivation of the tuber, including N levels. In this regard, the authors also describe the different practices in use for the improvement of NUE. However, I would suggest to the authors to add some additional information about the cultivars used in this work. Is there a specific reason related to the use of these selected CVs? Were they chosen for economical, nutritional, genetic or what? This information should be added to the last part of the introduction.

**Response: Thank you for your comments. The utilization efficiency of N of “Yanshu 4”, “Xiabodi” (cv. Shepody) and “Chunshu 4” potatoes are quite different in the production practice. Therefore, these three potato varieties are used as experimental objects.**

MATERIALS and METHODS: sometimes the methods are not well described or reported. I suppose the authors used qRT-PCR to validate the RNA-seq analysis. A couple of times they used PCR (also in the abstract) not qRT-PCR. I would suggest to add EC numbers for nitrate reductase and glutamine synthetase and also to provide some additional information about enzymatic activity measurements, although a kit was used. I don’t understand the unit used for enzyme activity calculation (see Figure 1). Moreover, what does it mean “root activity”?

**Response: Thank you for your comments. All the PCR mentioned in MATERIALS and METHODS should be qRT-PCR. We have revised the related parts. The EC number for nitrate reductase and glutamine synthetase was provide as required. The function of root activity was added in the method section. For the unit, we revised it in the newly submitted manuscript.**

In the RESULTS section, several information are missed, and must be implemented. I think it is not strictly necessary reporting numerical values in the manuscript text if present in the tables, however, at least the variation of the trend must be described (e.g. % of the change among the different CVs or between the two treatments). On the other hand, when data are expressed as Figures, it is necessary to report in the text the most important and representative numerical values (means ± standard deviation), and describe the changes among CVs and treatments.

**Response: Thank you for your comments. We added some main information to the manuscript. But it is unrealistic to fully describe the range of increase or decrease, especially the content in the table. We have two treatments for 3 varieties, so the range of variation is diverse. Even the changing trends of different varieties are also different between different treatments. Therefore, we only added the main content.**

The DISCUSSION is well motivated, but is limited only to the data obtained in this work. This part must be expanded. I suggest to introduce a short initial part, in which the importance of nutritional and economical aspects of potato are discussed in relation with the three different CVs employed. In addition, the importance of N, and its deficiency in plant nutrition has to be introduced.

**Response: Thank you for your comments. We added the importance of nutritional and economical aspects of potato. The utilization efficiency of N of “Yanshu 4”, “Xiabodi” (cv. Shepody) and “Chunshu 4” potatoes are quite different in the production practice. Therefore, these three potato varieties are used as experimental objects. The importance of N was also discussed in the newly submitted manuscript.**

The CONCLUSIONS are too synthetic and don’t highlight the obtained results. I suggest to expand also this section by explaining the importance of the research and the future applications.

**Response: Thank you for your comments. We rewrote our conclusions, summarizing the important results of this study and the importance of this study for potato breeding.**

LEGENDS FOR FIGURES: they must be improved. The authors should report as much information as possible. For example the statistical treatment used and the meaning of the letters on the bars (Figure 1). The legend must be clear for the reade

**Response: Thank you for your comments. All the legends of figures and tables were improved.**
