# Supplementary material for: Transcriptome analysis reveals Nitrogen deficiency induced alterations in leaf and root of three cultivars of potato (Solanum tuberosum L.)
Source: PLoS One. 2020 Oct 29;15(10):e0240662. doi: 10.1371/journal.pone.0240662 (PMC7595393; doi:10.1371/journal.pone.0240662)
Supplement: Attachment — Submitted filename: Response.docx [file pone.0240662.s005.docx]

Dear editor:

Thank you for your letter and for the reviewers’ comments concerning our manuscript entitled “Transcriptome analysis reveals Nitrogen deficiency induced alterations in leaf and root of three cultivars of potato (Solanum tuberosum L.)”. Those comments are all valuable. We have studied comments carefully and made corrections.

The revised manuscript is highlighted in Tracked Changes version.

**Point-by-point response to the reviewer’s comments**

Response:

This paper by Zhang et al. investigates the association of transcriptomic profiles with N deficiency using 3 potato cultivars. This study will have a bigger impact if the authors discuss the implications of their results in relation to genetic variability which was barely discussed.

Response: Thank you for your comments. The discussion section was improved. At the genetic level, the different expression patterns of genes in response to N deficiency were determined by gene diversity, which might also be the root cause of different varieties of potatoes having different responses to N deficiency. How to make good use of these excellent genetic resources for cross breeding was very worthy of our future research, and “Yanshu 4” might be an excellent candidate breeding resource. This part of the content has been added in the discussion.

Similarly, it is best to include in their discussion on how their results differ or add up to those already contributed by Jozefowicz et al. (2017) and Tiwari et al. (2020).

Response: Thank you for your comments. Jozefowicz’s research showed similar results with our findings, but the difference is that the N deficiency has a more extensive influence on the gene expression profile than protein level of high-resistant varieties. The experimental design of Tiwari’s research is also different from this study, but some of the results are consistent. In the discussion, we added a part of the content to explain the difference between this study and previous studies, but more importantly, discuss the possible reasons that caused the results of this study

Moreover, the conclusion needs to be further improved and expounded as it remains shallow reiterating their findings but without value/importance.

Response: Thank you for your comments. We have condensed the conclusion part to make the conclusion more prominent.

Statistical analyses only mentioned unpaired t-test and two-way ANOVA in the Methods while there is a plethora of statistical analyses (Holm- Sidak, Tukey’s Test, etc) which are more appropriate for this study.

Response: Thank you for your comments. The data were analyzed using the ANOVA test followed by Tukey post-hoc. We missed the test method in the previous version, this time we added it.

How was the correlation analysis done to conclude positive/negative correlation?
Response: Thank you for your comments. Correlation were analysis by Pearson correlation analysis.

The paper needs to be presented in a more intelligible manner (scientific jargon not just standard English). The following lines/sentences needs to be rephrased or improved:
Lines: 49, 69, 74-75, 94-97, 102-104, 179-188, 199-201, 208-214, 220-225,228-231, 237-239, 243-251,270-271, 275-276, 284-288, 309-311, 354, 370, 374-376
Response: Thank you for your comments. All the mentioned parts were revised as required.

Others:
Line 28 what is correlation relationship? Isn’t this positive or negative correlation?
Line 79 Delete “in April 2017”
Line 110 RNA-seq analysis using ___ (brand & model of equipment)
Line 111 delete as YNa, YNb …..
Line 216 Delete the terms “statistics of the”
Tables 1 and 2 need to be presented as graphs in figure with error bars representing SD.
Line 251 N containing fertilizer (not contained)
Line 299-300 what is PGSC… and PGSC…?
Line 323 start new paragraph with, “Nitrogen is the nutrient…
Line 330 what is N-colplete fertilization?
Line 331 start new paragraph with, “Studies have shown ….

Response: Thank you for your comments. The mentioned parts were all revised as required.

Reviewer #2: The manuscript has been improved and most part of my comments were properly addressed.
However, some revision is still necessary.
Line 28: GS and NR must be written with the full names as well. The same for StGDH, StGS and StCA (Line 30) and MFS members, like StNRT2.4, StNRT2.5 and StNRT2.7 (Line 33)
Line 50. Its tuber is used not tuber of which
Line 116. I would write the anthrone method and Coomassie Brilliant Blue G-250 dye-binding method were used…
Line 117. To detect not to detected
Line 118. I think that it is better to report the plural. Soluble sugars and soluble proteins.
Line 125: which kind of phosphate buffer? K or Na? I think the following sentence is unclear: Then the color reaction is carried out, and finally the absorbance is measured to determine the enzyme activity. Better to rewrite the sentence and use the past tense.
Line 132. RNAseq analysis since seq is already sequencing
Line 164. CTAB: report the full name as well. Methods should be singular, so method
Line 207. Sugars and proteins, not sugar and protein
Line 243. Respectively, not respectiverly
Line 261. Figure legend. Bar not bat
Line 270. DEG not DEGs
Line 318. N metabolism not N metabolisms
Line 392. N-completed not N-colplete
Line 416. DEG not DEGs
Response: Thank you for your approval and comments. All the mentioned parts in the manuscript was revised as required.

Supplementary
Table S1: qRT-PCR not PCR

Response: Thank you for your comments. We revised it as required.
