# Supplementary material for: Transcriptome analysis reveals Nitrogen deficiency induced alterations in leaf and root of three cultivars of potato (Solanum tuberosum L.)
Source: PLoS One. 2020 Oct 29;15(10):e0240662. doi: 10.1371/journal.pone.0240662 (PMC7595393; doi:10.1371/journal.pone.0240662)
Supplement: Attachment — Submitted filename: Response.docx [file pone.0240662.s006.docx]

Dear editor:

Thank you for your letter and for the reviewers’ comments concerning our manuscript entitled “Transcriptome analysis reveals Nitrogen deficiency induced alterations in leaf and root of three cultivars of potato (Solanum tuberosum L.)”. Those comments are all valuable. We have studied comments carefully and made corrections.

The revised manuscript is highlighted in Tracked Changes version.

**Point-by-point response to the reviewer’s comments**

**Review Comments to the Author**

Reviewer #2: (No Response)

Reviewer #3: Overall the paper is well-written, and provides valuable insight on transcriptome analysis reveals N-deficiency in three cultivars of potato (Solanum tuberosum L.). Below are suggestions and comments to improve the clarity and message of the manuscript.

>1.The authors choose three cultivars potato, Yanshu 4, Xiabodi and Chunshu 4. How did the authors decide three cultivars? From cultivar resources?

There was different genetic background in three cultivars potato, the difference in the growth performance parameters may be not only affected by N-related genes. So, the hybrids offspring of two different varieties were more reasonable.

**Response:** Thank you for your comments. In the production practice, we found that the utilization efficiency of N of “Yanshu 4”, “Xiabodi” (cv. Shepody) and “Chunshu 4” potatoes are quite different in the production practice. According to production experience, under the condition of sufficient nitrogen fertilizer, “Yanshu 4” was a high-absorption but low-utilization potato, “Xiabodi” was a medium-absorption and medium-utilization potato, and “Chunshu 4” was a low-absorption but high-utilization potato (lines 72-75). The aim of this study was to reveal the transcriptional responses of different cultivars to N deficiency under different conditions.

In addition, you encourage us to hybrid offspring of two different varieties, this is very important for our follow-up study. We are willing to accept your opinion to carry out hybridization experiment and reveal the location of functional genes and QTLs associated with the N-utilization efficiency.

Thanks again for the above comment and suggestion.


>2. Unclear statistics: you mention three plants for each replicate have been used, the number of n should be used for statistical evaluation, which tests were applied in legends?
**Response:** Thank you for your comments. We added the number of replicates in the statistical analysis section. Also, the tests were also added in the legends of Figure 1, 2 and 3.

>3. In fig4 C D, why the DEGs were less under N-deficiency condition in cultivars. There may be a lot frontloaded genes in cultivars.
Reference: Barshis etal, 2013 “Genomic basis for coral resilience to climate change”
**Response:** Thank you for your comments. We accept your suggestion and discuss it in the revised manuscript (Discussion section, lines 361-362).

>4. In RNA-seq analysis: why choose the second leaf? not flag leaf? And in RT-qPCR, which leaf was used, growth stage?
**Response:** Thank you for your comments. Because of individual differences, the flag leaf size is different. To avoid the impact of this difference, we used the second leaf. For qRT-PCR analysis, the second leaf was also used. Samples were collected at bud stage (line 149).

>5. In Fig6 and 7, It would be two reasons about the differential response and regulation of in three cultivars of potato, promoter or copy number. The expression of one gene was not usually compared among different cultivars by RT-qPCR.

**Response:** Thank you for your comments. We added the reason in discussion section. Also, we have modified the figures to reflect intra species comparisons rather than inter variety comparisons (lines 379-379). Thank you again for your comments, which is very helpful to improve our article.
